# Harmonic Focus Versus Conventional Electrocautery for Femoral Artery Exposure: a “Direct” Comparison on the Same Patients

**Published:** 2020-02-20

**Authors:** M Maisto, L Ferrante, AM Giribono, M Sodo, M Panagrosso, A Peluso, L del Guercio, UM Bracale

**Affiliations:** 1 Vascular Surgery Unit, Department of Public Health, University Federico II of Naples, Naples, Italy; 2 General Surgery Unit, Department of Public Health, University Federico II of Naples, Naples, Italy

**Keywords:** Focus Harmonic Scalpel, femoral artery, complications, abdominal aneurysm, endovascular repair

## Abstract

Surgical access complications during endovascular aneurysm repair (EVAR) are reported relatively frequent. HARMONIC FOCUS^®^ (HF; Ethicon Endo-Surgery Inc., Cincinnati, Ohio, USA) is a device developed to improve bleeding control and reduce heat-related damage stemming from surgical preparation.

The aim of this study was to evaluate outcomes and safety of HF versus conventional haemostasis with electrocautery, both techniques used in the same patient. Five patients developed bilateral wound’s thickening (13.9%) demonstrated at the CT scan, two of whom had no clinical manifestation while in three cases the thickening was associated with lymphocele (4.54%), 2 of which were in the side where the EC was used (5.5%), and 1 case (2.7%), in the HF applied side. One isolated lymphocele occurred at the left groin (2.7%) (tables n.2–3). A Fisher’s exact test was conducted between EC and HF on the occurrence of wound healing complications (3/36 for EC and 1/36 for HF) that resulted statistically significant at p<0.05. Focus Harmonic Scalpel has certain advantages than conventional haemostasis in avoiding surgical access complications.

## I. INTRODUCTION

The surgical exposure of femoral vessels is relatively easy to perform yet can still be affected by several complications of surgical wound healing. While endovascular aneurysm repair (EVAR) has decreased morbidity and mortality rates when compared to open surgical repair, nevertheless some complications resulting from the EVAR procedure still remain. Surgical access complications such as lymphoceles, lymphorrhoea, wound infection and dehiscence are reported in the literature with an incidence rate of 13%^1^.

HARMONIC FOCUS® (HF; Ethicon Endo-Surgery Inc., Cincinnati, Ohio, USA) is a device developed to improve bleeding control and reduce heat-related damage stemming from surgical preparation. Through the use of ultrasound energy, the HR cuts and coagulates soft tissue and vessels at much lower temperatures than radiofrequency-based devices, with minimal thermal damage to the surrounding tissues 2. Ultrasonic energy is an efficient alternative to traditional electrocautery as its combination of high frequency (55.5 kHz) and limited intensity make it a risk-free method for operators and extremely safe and reliable for the patient. By using lower temperatures (50–100°) HF® scissors also increase the safety of the surrounding tissue, like nerves and other vessels. It also doesn’t interfere with implantable devices such as defibrillators and pacemakers, very common in vascular surgery patients, as it doesn’t use electricity. Notwithstanding these advantages, there is a paucity of literature on the HR and so herein we report on our experience using both electrocautery (EC) and HF^®^ on the same patient in the surgical exposure of femoral vessels for EVAR to evaluate any significant differences in terms of the presence of wound healing complications in mid and long-term follow-ups.

## II. METHODS

Data of all patients who had undergone EVAR with surgical exposure of femoral vessels in the period from January 2016 to December 2017 were prospectively collected in a dedicated database and analyzed. End-points were to evaluate perioperative mortality and morbidity with a focus on groin complication. Clinical data such as age, sex and risk factors as hypertension, diabetes, obesity or cigarette smoking were collected (table n.1), and perioperative therapy was also considered.

Informed consent was obtained from all patients. All operations were performed by young vascular surgeons in training under the supervision of fixed staff expert surgeons.

All patients had a preoperative CT-scan. The surgical access to the femoral vessels was consistently obtained using the conventional EC [Force EZ C Electrosurgical unit (EC, Valleylab, Boulder, CO)] in the left groin and the HF in the right one. Both instruments were used at the same time in each patient to create a homogenous patient sample. The skin incision was always longitudinal. The diameter of the introducer used, depending on the type of device to be implanted, was between 12–20 French. Inguinal surgical preparation, such as the wound suture, was performed by different surgical residents. A clinical evaluation of the wound was performed in all patients after 7–10 days and a postoperative CT-angio 4 and 8 weeks after surgery. Continuous variables are expressed as means ± standard deviations and frequencies by percentages. Analyses of the differences between the two types of apparatus were performed using the Fisher’s exact test and a univariate and multivariate analysis to identify independent risk factors for groin complications. Statistical tests were performed using SPSS 21.0 for Windows (IBM Corp, Armonk, NY).

## III. RESULTS

Throughout the study period 36 patients (4 women and 32 men) who were affected by different types of aorta aneurysms were treated with EVAR with surgical access through the femoral vessels (table n.2). Patients were also stratified for cardiovascular risk factors such as hypertension, diabetes, obesity or cigarette smoking (table n.3).

In all cases an antibiotic prophylaxis using 2 gr of Cephazolin before 30 minutes was administered before skin incision. At the time of placement, the introducers patients received 70–100 units/kg of heparin. Table n.1 lists the types of stent-grafts used.

Three patients received a “chimney EVAR treatment” (8.3%) under general anesthesia while the others received a loco-regional epidural anaesthesia (92.7%).

Five patients developed bilateral wound’s thickening (13.9%) demonstrated at the CT scan, two of whom had no clinical manifestation while in three cases the thickening was associated with lymphocele (4.54%), 2 of which were in the side where the EC was used (5.5%), and 1 case (2.7%), in the HF applied side. One isolated lymphocele occurred at the left groin (2.7%) (tables n.2–3). A Fisher’s exact test was conducted between EC and HF on the occurrence of wound healing complications (3/36 for EC and 1/36 for HF) that resulted statistically significant at p<0.05.

## IV. DISCUSSION

Surgical cut-down for EVAR access has been the standard approach for common femoral artery exposure. Although a relatively easy surgical approach, EVAR can still be affected by several complications, particularly in cases of redo access or scar tissue^3^. Nowadays EVAR is preferably performed through a total percutaneous approach when feasible. The aim of this study was to investigate the safety and efficacy of HF compared to EC in the same patients, simultaneously in real time Traditionally hemostasis is obtained by clamping and tying the vessels with or without electrocautery. The EC uses temperatures as high as 400 °C to obtain the carbonization of tissues. The newer generation of energy devices used for hemostasis deliver more focused thermal energy and reduce the risk of collateral tissue injury. The ultrasonic system is able to give optimal coagulation at relatively “cold” temperatures compared with electrocautery. These devices are multifunctional, capable of sealing, blunt-dissecting, grasping and dividing tissue, thereby carrying out surgical incisions in a more efficient manner. The two most commonly used energy devices are the ultrasonic coagulation shear device and the electrothermal bipolar vessel sealer 4–6.

Some authors have reported HF’s efficacy in performing thyroidectomies in terms of reduction of surgical time and other postoperative outcomes. Maeda et al.^7^ suggested that the duration of surgical time was significantly reduced using HF^®^ in an open thyroidectomy as well as intraoperative blood loss. Postoperative complications were also decreased. They assumed the reduction in hospitalization time was related to a reduction in surgical-site infections and the particular shape of the HF^®^ helped reduce adverse effects such as recurrent laringeal nerve paralysis. This is likely attributable to the fact that the tissues could be detached, coagulated and dissected with the HF^®^ in a continuous operation without the need to change instruments. Some studies 8–9 compared the HF^®^ to EC in open thyroidectomy and reported that the main advantage of using HF^®^ in a thyroidectomy was the reduced procedure time.

The most frequent complication of surgical femoral access is the lymphocele. Treatment of lymphoceles complicating vascular procedures is controversial. Porcellini et al. 10 recommends a conservative approach to lymphoceles including bed rest, repeated aspirations and pressure dressings on an outpatient basis.

The use of HF in vascular surgery is still very limited and few non-randomized studies exist. Santin et al. 11 performed a retrospective non-randomized study on the use of HF^®^ in inguinal exposure in EVAR, but the choice whether to use HF^®^ or EC seems to have been an arbitrary one. In our study the results of HF^®^ were similar to those reported by Satin, as corroborated by the perspective nature of the study. Moreover, by using two devices on the same patient at the same time we avoided the influence of different therapies or risk factors such as cigarette smoking, hypertension and obesity, widely represented in our population.

The risk of surrounding tissue injury was lessened with the HF since the amount of heat generated was lower than that required by EC and no electric current passed through the patient’s body - an important characteristic of the HF device - since many of our patients have implantable pacemakers or defibrillators which makes them unsuitable for EC^11^.

Some limits of the study were the small number of patients due to extensive percutaneous access to the femoral vessels which affected patient recruitment.

Technical aspects might also influence the results since the preparation of the femoral vessels was performed by different surgeons and so the experience of the operator could have a role in the presentation of wound complication however this cannot be ascertained from the data we collected. Therefore, a more refined surgical technique and familiarity with the instrument are required to avoid unwanted surrounding tissues damage and more conclusive results.

## V. CONCLUSION

In conclusion, despite the limited number of patients treated and a possible bias regarding the different operating surgeons, our experience resulted in a statistically significant difference between HF and EC in both complication and morbidity rates. Further studies with a larger sample size are needed to demonstrate the advantages of HF in accessing the femoral vessels.

## Figures and Tables

**Table n. 1:** Risk factors, Type of Aneurysm, Implantend Stent-grafts

Risk Factors	n (N =36)
Smoke	27 (75.0%)
Hypertension	17 (47.2%)
Diabetes	5 (13.8%)
Obesity (BMI>30)	14 (38.8%)
**Type of Aneurysm**	
Infrarenal	29 (80.5%)
Juxtarenal	1 (2.7%)
Aortoiliac	5 (13.8%)
Hypogastric	1 (2.7%)
**Implanted Stentgrafts**	
Endurant (Medtronic)	12 (33.3%)
Excluder C3 (Gore)	10 (27.7%)
E-Tegra (Jotec)	10 (27.7%)
Aorfix (Lombard Medical)	2 (5.5%)
Treovance (Bolton Medical)	2 (5.5%)

**Table n. 2:** Wound healing complications at clinical and CT check

Wound healing complications at clinical and CT check	n (N=36)
Bilateral thickening	5 (13.9%)
Thickening + Right lymphocele	1 (2.7%)
Left lymphocele	2 (5.5%)
Isolated left lymphocele	1 (2.7%)

**Table n.3:** Complications at surgical site with HF and EC

Complications at surgical site	HF	EC
Bilateral thickening	5 (13.8%)	5 (13.8%)
Associated lymphocele	1 (2.7%)	2 (5.5%)
Isolated lymphocele	0	1 (2.7%)
